# Virtual reality and live scenario simulation: options for training medical students in mass casualty incident triage

**DOI:** 10.1186/cc11086

**Published:** 2012-03-20

**Authors:** PL Ingrassia, L Ragazzoni, L Carenzo, FL Barra, D Colombo, G Gugliotta, F Della Corte

**Affiliations:** 1 CRIMEDIM Research Center in Disaster and Emergency Medicine, Novara, Italy

## Introduction

Multicasualty triage is the process of establishing the priority of care among casualties in disaster management. Recent mass casualty incidents (MCI) revealed that health personnel are unfamiliar with the triage protocols. The objective of this study is to compare the relative impact of two simulation-based methods for training medical students in mass casualty triage using the Simple Triage and Rapid Treatment (START) algorithm.

## Methods

A prospective randomized controlled longitudinal study. Medical students enrolled in the emergency medicine course were randomized into two groups (A and B). On day 1, group A students were exposed to a virtual reality (VR) scenario and group B students were exposed to a live scenario (LS), both exercises aiming at triaging 10 victims in a limited period of time (30 seconds/victim). On day 2 all students attended a 2-hour lecture about medical disaster management and START. On day 3 group A and B students were exposed to a LS and to a VR scenario respectively. The vital signs and clinical condition of the 10 victims were identical in the two scenarios. Ability of the groups to manage a simulated triage scenario was then compared (times and triage accuracy).

## Results

Groups A and B were composed of 25 and 28 students respectively. During day 1 group A LS triage accuracy was 58%, while the average time to assess all patients was 4 minutes 28 seconds. The group B VR scenario triage accuracy was 52%, while the average time to complete the assessment was 5 minutes 18 seconds. During day 3 the triage accuracy for group A VR simulation was 92%, while the average time was 3 minutes 53 seconds. Group B triage accuracy during the LS was 84%, with an average time of 3 minutes 25 seconds. Triage scores improved significantly during day 3 (*P *< 0.001) in the two groups. The time to complete each scenario decreased significantly from day 1 to day 3.

## Conclusion

The study demonstrates that the training course generates significant improvement in triage accuracy and speed. It also reveals that VR simulation compared to live exercises has equivalent results in prompting critical decisions in mass casualty drills. In the beginning the average time to complete the VR scenario was higher than the LS. This could be due to the fact that on day 1 very detailed VR victims created a higher challenge for untaught students. However, the higher triage accuracy recorded at the end of day 3 in VR could be explained by a lower stress level compared to the LS, which could be creating a more stressful environment in taught students.

